# Exploring the factors contributing to parent stress symptoms during the COVID‐19 pandemic in Europe: An ABC‐X model approach

**DOI:** 10.1111/famp.13063

**Published:** 2024-09-27

**Authors:** Anis Ben Brik, Yunqi Wang

**Affiliations:** ^1^ College of Public Policy Hamad Bin Khalifa University Ar‐Rayyan Qatar; ^2^ Department of Child, Youth and Family Studies University of Nebraska–Lincoln Lincoln Nebraska USA

**Keywords:** ABC‐X model, Europe, family relationships, parental stress, relationship satisfaction, resilience beliefs

## Abstract

The COVID‐19 pandemic has had a significant impact on the mental health and well‐being of families worldwide, with parents particularly at risk for stress and other psychological symptoms. In this study, we sought to understand the factors contributing to parent stress symptoms during the early stages of the pandemic in 23 European countries (*N* = 40,138) using the double ABC‐X model. We examined whether the relationship between stressor pile‐up and perceived stress was mediated by family satisfaction and relationship satisfaction and whether family resiliency beliefs impacted these mediated relationships. Our results showed a direct association between stressor pile‐up and parent stress symptoms, but we did not find evidence for the mediating role of family satisfaction or relationship satisfaction in this relationship. We also found that family resiliency beliefs did not moderate the indirect effects of family satisfaction and relationship satisfaction on parent stress symptoms. These findings suggest that the ABC‐X model may not fully capture the processes affecting parents' experience of stress during the pandemic and that alternative models such as the vulnerability‐stress‐adaptation model may be more relevant. Future research should also consider the potential negative impact of resiliency beliefs on mental health and other risk and protective factors such as self‐compassion.

The COVID‐19 pandemic has had a significant impact on the daily lives of parents and children in Europe. Many countries in Europe, including Italy, Spain, and France, implemented lockdowns and quarantines in an attempt to slow the spread of the disease (European Centre for Disease Prevention and Control, 2021). These measures have had a range of impacts on parents' mental health, with research indicating an increase in psychological symptoms and distress during lockdowns and quarantines (Brooks et al., 2020; Mazza et al., 2020). For instance, Italian parents reported lower levels of well‐being, self‐control, and anxiety (Cusinato et al., 2020). In one Spanish sample, nearly half (44.1%) of the respondents reported symptoms of depression, while a third (32.4%) reported symptoms of anxiety (Salari et al., 2020). Parents in Europe have reported increased stress while managing childcare, schoolwork, and their own jobs and careers during the pandemic (Calvano et al., 2021; O'Sullivan et al., 2021). These stressors can have negative effects on individual well‐being and can worsen pre‐existing psychological disorders or trauma (Calvano et al., 2021; Fernández‐Aranda et al., 2020; Mazza et al., 2020).

Unique challenges were presented for parents and families during the pandemic, including economic uncertainty, concerns about physical health, and difficulties with homeschooling and managing children's isolation from friends and teachers (Fontanesi et al., 2020). In addition, parents have had to cope with the added burden of communicating with their children about the pandemic in an age‐appropriate and calming way (Calvano et al., 2021; O'Sullivan et al., 2021). Although evidence suggests widespread negative impacts of the pandemic on parents' mental health, few studies have explored family mechanisms through which pandemic stressors impact mental health. Therefore, the current study aims to explore the factors impacting parental stress using the double ABC‐X model during the early stage of the pandemic in the European families. Understanding parental stress through a family dynamics lens is crucial for both clinical practice and societal well‐being. Identifying the mechanisms through multiple stressors across contexts that influence stress levels could contribute to more effective therapeutic interventions and better mental health outcomes for parents. Therapists and counselors can develop targeted approaches to strengthen positive family dynamics and mitigate stressors, ultimately improving the overall mental health of the family unit. On the societal level, recognizing the stress dynamics in families is important for informing public health policies and community programs. Policymakers may consider addressing factors to promote family relationships, such as financial strain, work‐life balance, and social support. These practices in the long run could help mitigate the stress‐related issues, promote youth development, and foster more resilient communities, especially in the face of adversity.

## Theoretical framework

The Double ABC‐X model of family stress and adaptation (McCubbin & Patterson, 1983; Rosino, 2016) can guide this study. This model, based on the social system theory, views the family as a system that must maintain equilibrium to meet its members' needs. Stressful events, such as the COVID‐19 pandemic, can disrupt family equilibrium and lead to negative outcomes unless families adopt practices that support well‐being, such as providing social and emotional support and utilizing available resources (Burr & Klein, 1994). The Double ABCX model identifies several predictor variables that may impact the outcome of a crisis or stressor on a family's functioning and well‐being. These variables include (a) the accumulation of stressors after the crisis onset, known as stressor pile‐up, (b) the family's existing adaptive resources, and (c) the family members' perceptions and coherence of the stressor. The outcome variable (x) represents the extent to which the stressor causes a new crisis that threatens the family's functioning and well‐being (Rosino, 2016). In the context of the pandemic, stressor pile‐up may include the direct consequences of the virus such as changes in caretaking and childcare responsibilities, job‐related challenges, and financial strains. Pre‐existing challenges in the family, such as educational, physical, or mental health issues, may also be exacerbated by the pandemic and contribute to the accumulation of stressors (Boettcher et al., 2021; Slopen et al., 2018). Variable (b) in the Double ABC‐X model of family stress and adaptation refers to the family's adaptive resources, which can influence the impact of stress on the family's outcomes (Boss, 2002). Adaptive resources within the family environment may include family satisfaction and romantic relationship satisfaction (Stavropoulos et al., 2015). Family satisfaction, or an individual's evaluation of their family relationships and communication, has been linked to decreased satisfaction in the face of stress (Price et al., 2017).

During the pandemic, the studies have found that pandemic‐related stressors negatively impact family satisfaction, particularly among families with minor children (Mohring et al., 2021; Rudolph & Zacher, 2021). Decreased family satisfaction has also been linked to poorer mental health outcomes for parents (Huebener et al., 2021; Mohring et al., 2021). The pandemic has also had a negative impact on romantic relationship satisfaction, which can affect family members' mental health (Randall & Bodenmann, 2009). External stressors such as financial problems and health issues can strain relationships and exacerbate existing problems (Cohan, 2010), leading to decreased satisfaction and poor mental health outcomes (Braithwaite & Holt‐Lunstad, 2017). There is a link between relationship satisfaction and mental health, with the strongest effects seen when mental health is affected by low relationship satisfaction (Braithwaite & Holt‐Lunstad, 2017). The risk of romantic relationship conflict is also increased during the pandemic due to the significant stress many families are experiencing (Prime et al., 2020; Schmid et al., 2021).

Variable cC in the Double ABC‐X model refers to how a family understands and cohesively deals with a stressful situation. This concept has been studied in various ways, including how individuals interpret the severity of the stressor and the family's ability to effectively handle it. One factor that may help protect families from mental health issues during the pandemic is resilience. Resilience beliefs can impact mental health during challenging times by shaping how individuals perceive the event and maintain a positive outlook, despite current difficulties (Walsh, 1996). When families fit challenges caused by disasters into their existing resilience beliefs (or adjust them) in a way that promotes unity and coherence, it can promote positive adaptation in disaster situations (Walsh, 2021). Therefore, families who view the pandemic and related stressors as temporary, manageable, and not caused by personal faults, avoid catastrophic thinking and dwelling on worst‐case scenarios, and maintain hope, a focus on the future, and the belief that adversity can lead to growth, may be less likely to experience poor mental health (Walsh, 2021). Recent research using the Double ABC‐X model has found that both adaptive resources (bB variables) and perceptions and coherence (cC variables) play a role in the connection between stressor accumulation and adaptation, such as mental health, stress, health‐related quality of life, and family dysfunction. For example, Boettcher et al. (2021) used a parallel multiple mediation model to examine how mothers' adaptation to a rare congenital surgical disease in their children was influenced by family functioning, social support, and perceived stress. Similarly, Frishman et al. (2017) looked at caregiver quality of life in caregivers for children with childhood‐onset dystrophinopathy and found that the relationship between stressor accumulation was mediated by global perceived social support, supportive family relationships, and perceived stress and control. According to the ABC‐X model, a family's available adaptive resources and their perceptions of the stressor jointly determine the family's adaptation to the stressful event (Rosino, 2016). A moderated mediation analytic approach can be used to test the possibility of a mediated relationship between variables. In a moderated mediation, the mechanism through which an independent variable affects a dependent variable is moderated by a fourth variable, such that the mediating (indirect) effect is different at different values of the moderator (Hayes, 2018). Moderated mediation tests conditional indirect effects, evaluating whether a moderating variable influences the mediated relationship between an independent and dependent variable.

## Present study

**FIGURE 1:**
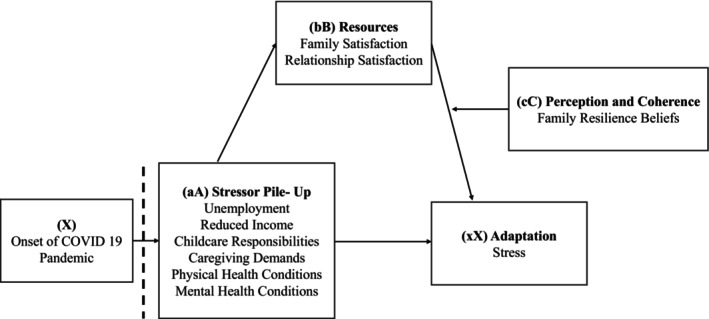
Conceptual model.

The primary goal of this study was to explore factors indicated in the double ABC‐X model that explain parent stress symptoms early in the pandemic in 23 European countries. Specifically, we explored whether the relationship between the stressor pileup represented by, “aA” and adaptation, “xX” is mediated by resources available, and “bB”. Additionally, we explored whether this mediation is impacted by perceptions, “cC”. A graphic representation of our hypotheses is presented in Figure 1. The main predictor was the Stressor Pileup, while family satisfaction and relational satisfaction were mediators. We predicted family resiliency beliefs as a moderator, with parent stress symptoms as the dependent variable. Age, gender, education attainment, and employment status were used as covariates in the analysis. Our hypothesis predicting a second stage moderating mediation model where family resiliency beliefs moderated the second stage indirect paths of family satisfaction and relationship satisfaction, creating conditional indirect effects.

## METHODS

### Participants

The data for this study were drawn from the COVID‐19 Family Life Study (Ben Brik, 2020), which explored the effects of the coronavirus pandemic on families in 72 countries from every inhabited continent, representing more than 75% of the world's population. The current study specifically utilized data from a European sample of 23 countries: Germany (*N* = 2376), France (*N* = 2210), Italy (*N* = 2479), Spain (*N* = 2052), Portugal (*N* = 1267), Greece (*N* = 1876), the Netherlands (*N* = 2072), Poland (*N* = 1575), Hungary (*N* = 1490), Romania (*N* = 1694), Croatia (*N* = 1837), Estonia (*N* = 1552), Lithuania (*N* = 1662), Bulgaria (*N* = 1397), Austria (*N* = 1791), Belgium (*N* = 1956), Latvia (*N* = 1446), Ireland (*N* = 1786), Sweden (*N* = 1341), Slovakia (*N* = 1508), Norway (*N* = 1709), the Czech Republic (*N* = 1632), and Slovenia (*N* = 1430). The sample includes 40,138 participants, but the analytical sample for this study only includes a subset of participants who are married or living with a romantic partner (*N* = 37,421). These individuals were selected from the panel to be nationally representative in terms of age, gender, education, and marital status (i.e., quota sample) of parents with children under 18. Table 1 displays the descriptive statistics of the sample. On average, slightly fewer than half of the participants in the four countries are parents aged 30–44 years old, more than half are women, and most have educational qualifications. A little over half of the participants are full‐time employed.

**TABLE 1:** Demographic characteristics of the sample (*N* = 37,421).

Baseline characteristic	Full sample	
	*n*	%
*Gender*		
Male	16,690	44.6
Female	20,731	55.4
*Age*		
<29	636	1.7
30–44	20,320	54.3
45–64	14,145	37.8
+65	2320	6.2
*Educational attainment*		
Less than high school	486	1.3
High school diploma	3069	8.2
Some college	8532	22.8
College degree	17,401	46.5
Post‐graduate	7933	21.2
*Employment status*		
Self‐employed	2208	5.9
Part‐time employment	4154	11.1
Full‐time employment	21,779	58.2
Unable to work due to disability	561	1.5
Homemaker/Stay at home parent	5763	15.4
Unemployed and seeking work	2395	6.4
Retired	561	1.5

### Procedure

Participants in the 23 European countries were recruited through convenience sampling for a self‐administered survey between April and September of 2020. The survey was distributed via social media, and email databases of the European Large Families Confederation (ELFAC), the German Society for Housekeeping, the International Association for Home Economics (IFHE), the Alliance for Integrated Care (AfIC) in Greece, the National (Swedish) Association of Stay‐at‐Home Parents (HARO), the Association Obitelji 3plus in Croatia, and the Association of Large Families in Romania (ASFANU). The survey was conducted on the SurveyMonkey platform and data were collected for ~4–5 weeks in each country after the study design and materials were approved by Hamas Bin Khalifa University (QBRI‐IRB 2020–06‐021). The questionnaire was available in 21 languages, including English, German, French, Italian, Spanish, Portuguese, Greek, Dutch, Polish, Hungarian, Romanian, Croatian, Estonian, Lithuanian, Bulgarian, Latvian, Swedish, Slovak, Norwegian, Czech, and Slovene. These translations were created using the translation back‐translation method (Brislin, 1970), with two independent translators working on each language version to ensure cultural adaptation, clarity, common language use, and conceptual equivalence.

### Measures

Stressor Pile‐Up (aA factor). Following procedures described in the published studies testing the ABC‐X model, a composite variable reflecting stressor pile‐up was created by summing dichotomous variables together (Boettcher et al., 2021; Paynter et al., 2013). The stressor pile‐up count was based on the sum of the following items: (a) lost employment due to the COVID‐19 pandemic (no = 0, yes = 1), (b) experienced a reduction income due to the COVID‐19 pandemic (no = 0, yes = 1), (c) had to alter work arrangements due to different childcare responsibilities imposed by the COVID‐19 pandemic (no = 0, yes = 1), (d) caring for a child with special educational needs (no = 0, yes = 1), (e) responsible for providing care for an elderly relative or friend (no = 0, yes = 1), (f) responsible for providing care to an individual with a chronic health condition or a disability (no = 0, yes = 1), (g) self or other member of household has one or more chronic physical health conditions (no = 0, yes = 1), and (h) self or other member of household has one or more chronic mental health conditions (no = 0, yes = 1). The resulting composite variable had a possible range of 0–8. The scale has been translated and culturally adapted in 21 languages using the forward–backward translation process (Brislin, 1970). A pilot study was conducted with 50 randomly selected participants in each country to assess the scale's internal consistency and reliability using Cronbach's alpha. The scale was found to have good internal reliability, with Cronbach's alpha coefficients ranging from 0.78 to 0.94 (see Table S1).

### Family satisfaction (bB factor)

The bB factor was assessed using the Family Satisfaction Scale (FSS), developed by Olson (1995) in relation to the Circumplex model. The scale assesses the degree of satisfaction with aspects related to family cohesion, flexibility, and functioning such as decision‐making, family boundaries, and rules. The current version of the Family Satisfaction Scale contains 10 items on a 5‐point Likert‐type scale ranging from 1 = Very Dissatisfied to 5 = Extremely Satisfied. Example items included, *family members are involved in each other's lives*, *family members are supportive of each other during difficult times*, and *family members like to spend some of their free time with each other*. The scale is scored by summing the 10 items so that higher scores indicate more satisfaction. The potential range was 10–50 and the observed range was 10–50. In this study, we used the original English version of the Family Strengths Scale, as well as validated versions in Italian (Baiocco et al., 2012), Greek (Koutra et al., 2012), Hungarian (Mirnics et al., 2010), Polish (Margasiński, 2014), Portuguese (Gomes et al., 2017), Spanish (Sanz et al., 2002), and Romanian (Rada, 2017). The scale was translated using the back‐translation method and culturally adapted for use in languages without validated versions (German, French, Dutch, Croatian, Estonian, Lithuanian, Bulgarian, Latvian, Swedish, Slovak, Czech, and Slovene). Cronbach's alpha coefficients for the different language versions ranged from 0.78 to 0.94, indicating good internal reliability. These findings align with previous research on the original English version of the FSS (Olson, 2011).

### Relationship satisfaction (bB factor)

The bB factor was assessed using The ENRICH Marital Satisfaction Scale (EMS), developed by Fowers and Olson (1993). The scale is a brief measure of romantic relationship quality (Fowers & Olson, 1993). This instrument includes items covering fundamental aspects of relationships including communication, conflict resolution, roles, financial concerns, leisure time, sexual relationship, parenting, family and friends, and religion. Respondents indicate the extent of their agreement with 15 statements using a 5‐point Likert scale with 1 = *strongly disagree*, 2 = *disagree*, 3 = *neither agree nor disagree*, 4 = *agree*, and 5 = *strongly agree*. Example items included, *My partner and I understand each other perfectly*, *I am very happy about how we make decisions and resolve conflicts* and *I am very happy with how we manage our leisure activities and the time we spend together*. An overall individual EMS score is calculated by using the Idealistic Distortion percentile score to correct the Marital Satisfaction percentile score downward on the basis of the degree to which the respondent portrays the marriage in an impossibly positive way, using the following formula in which PCT = percentile score for the Marital Satisfaction scale and ID = percentile score for the Idealistic Distortion scale: EMS score = PCT – [(0.40 × PCT) (ID × 0.01)]. In this study, we used the original English version of the EMS and validated translations in Spanish (Escriba‐Aguir & Artazcoz, 2010), Portuguese (Marques, 2001), and French (Vandeleur et al., 2003). We also developed translations for the German, Greek, Dutch, Polish, Hungarian, Romanian, Italian, Croatian, Estonian, Lithuanian, Bulgarian, Latvian, Swedish, Slovak, Norwegian, Czech, and Slovene versions through a process of forward–backward translation. The internal reliability of these translations, as measured by Cronbach's alpha, ranged from 0.78 to 0.94 and was consistent with previous validation studies (Fowers et al., 1996; Fowers & Olson, 1993).

### Family resilience beliefs (cC factor)

The cC factor was assed using the 10‐item Connor‐Davidson Resilience Scale (CD‐RISC‐10), developed by Connor and Davidson (2003). The instrument is comprised of ten of the original 25 items from the Connor‐Davidson Resilience Scale. It measures resilience and includes items regarding adaptability and stress‐management abilities. Example items included, *tend to bounce back after illness or hardship*, *When things look hopeless, I don't give up*, and *coping with stress strengthens*. A 5‐point Likert scale was used (0 = *not true at all*, 4 = *true all the time*). A respondent's total score can range from (0 = *not true at all*, 4 = *true all the time*), with higher scores indicating higher levels of resilience. This study used the original English version and validated translations of the CD‐RISC in 20 languages: German, French, Italian, Spanish, Portuguese, Greek, Dutch, Polish, Hungarian, Romanian, Croatian, Estonian, Lithuanian, Bulgarian, Latvian, Swedish, Slovak, Norwegian, Czech, and Slovene (Connor & Davidson, 2003). The scale demonstrated good internal consistency, with Cronbach's alpha coefficients ranging from 0.78 to 0.94 for the 20 translations. These results are consistent with the previous research on the original English version of the CD‐RISC (Connor & Davidson, 2003).

### Parent stress symptoms (xX factor)

The xX factor was assessed using the Depression, Anxiety and Stress Scale—21 Items (DASS‐21) developed by Norton (2007). The DASS‐21 is a widely used instrument for assessing depression, anxiety, and stress symptoms worldwide (Scholten et al., 2017) and is a condensed version of the DASS‐42 (Lovibond & Lovibond, 1995), which has been translated into 45 languages to date. The DASS‐21 has been validated in clinical samples (Antony et al., 1998; Lovibond & Lovibond, 1995). Following Norton (2007), we used the full 21‐item, with a response scale of 0 (*did not apply to me at all*) to 3 (*applied to me very much, or most of the time*), instead of breaking the scale into its constituent parts. Example items included, *I found it hard to wind down*, *I felt depressed and had no motivation*, and *I felt I had no desire for anything*. Scores for the DASS‐21 were calculated by summing the scores for the relevant items per scale; then, the DASS‐21 subscale total was multiplied by two to give the final score for categorization into: normal (0–9 for depression, 0–7 for anxiety, and 0–14 for stress), mild/moderate (10–20 for depression, 8–14 for anxiety, and 15–25 for stress), and severe/extremely severe (21+ for depression, 15+ for anxiety, and 26+ for stress; Lovibond & Lovibond, 1995). In this study, DASS‐21 was used in its original English version and in validated translations in German, French, Italian, Spanish, Portuguese, Greek, Dutch, Polish, Hungarian, Romanian, Croatian, Lithuanian, Latvian, Swedish, Slovak, Norwegian, Czech, and Slovene. Translations were also developed for Estonian and Bulgarian using the translation and back‐translation method (Brislin, 1970). The internal consistency of the Estonian and Bulgarian versions was 0.87 and 0.94, respectively, while the internal consistency of the other versions ranged from 0.78 to 0.94. These results are consistent with previous research on the original English version of the DASS‐21 (Lovibond & Lovibond, 1995).

### Statistical approach

SPSS 28 was used to conduct all statistical analyses. The preliminary analyses included data screening for outliers and to confirm that continuous variables adhered to a normal distribution (Tabachnick & Fidell, 2013). The values for skewness and kurtosis, as well as the results of tests for normality (i.e., Shapiro–Wilk test), were examined to ensure that the continuous study variables adhered to a normal distribution. All values were within acceptable ranges to infer normality.

The hypothesized moderated mediation model (see Figure 1) was tested in a single model using a bootstrapping approach to assess the significance of the indirect effects at differing levels of the moderator (Hayes, 2013). Stressor pile‐up was the predictor variable, with family satisfaction and relationship satisfaction as the mediators. Including both hypothesized variables in the mediation model simultaneously (versus estimating separate models for each variable) yields the estimates of the indirect and direct effects that are unique to each variable. The outcome variable was parent stress symptoms and family resilience beliefs were the proposed moderator. Moderated mediation analyses test the conditional indirect effect of a moderating variable (i.e., family resilience beliefs) on the relationship between a predictor (i.e., stressor pile‐up) and an outcome variable (i.e., stress) via potential mediators (i.e., family satisfaction and relationship satisfaction).

The PROCESS macro, model 14, v4 (Hayes, 2013) with bias‐corrected 95% confidence intervals was used to test the significance of the indirect (i.e., mediated) effects moderated by family resilience beliefs, i.e., conditional indirect effects. An advantage of this macro is that it implements the recommended bootstrapping procedures and automatically computes post‐hoc probing for moderating effects. The model was estimated using 5000 bootstrapped samples. To evaluate moderated mediation, the significance of the conditional indirect effect was estimated at the 16th, 50th, and 84th values of the moderators. Confirmation of moderated mediation was based on the index of moderated mediation (Hayes, 2015). Like traditional moderation analyses where a significant interaction suggests that the simple slopes are different from each other (Aiken et al., 1991), a significant index of moderated mediation indicates that the moderator is linearly related to the indirect effect and implies that the conditional indirect effects defined by the two different values of the moderator are statistically different. Significance of the index of moderated mediation (i.e., evidence of moderation of the indirect effects of the relations between stressor pile‐up and parent stress by family satisfaction and relationship satisfaction) is established when the bootstrap confidence interval for the index of moderated mediation does not include zero.

## RESULTS

### Descriptive statistics and correlation analysis

Descriptive statistics and bivariate correlations among the variables examined in this study are summarized in Table 2. Stressor pile‐up was positively associated with parent stress (r = 0.106, *p* < 0.01), and negatively associated with perceived resilience (r = −0.12, *p* < 0.01). The association between stressor pile‐up, family satisfaction and relationship satisfaction were positive but statistically non‐significant, respectively. In addition, higher family satisfaction was associated with lower relationship satisfaction (*r* = −0.128, *p* < 0.01), lower family resilience beliefs (*r* = −0.374, *p* < 0.001) and higher perceived stress (*r* = 0.294, *p* < 0.001). Furthermore, lower perceived stress was associated with both higher relationship satisfaction (*r* = −0.135, *p* < 0.001) and higher family resilience beliefs (*r* = −0.317, *p* < 0.001).

**TABLE 2:** Descriptive statistics and correlations among variables (*N* = 37,421).

Variable	Mean	SD	Range	1	2	3	4
Stressor pile‐up	4.62	1.79	0–8	_			
Family satisfaction	21.83	10.51	10–50	0.043	_		
Relationship satisfaction	46.19	10.08	9–81	−0.054	−0.128*	_	
Family resilience beliefs	27.04	11.88	0–40	−0.120*	−0.374**	0.198**	_
Stress	14.52	9.76	0–21	0.106**	0.294**	−0.135*	−0.317**

* *p* < 0.01.

** *p* < 0.001.

### Mediation analyses

After adjusting for various factors, including gender, age, race, educational attainment, and employment status, the mediation analysis (Table 3) revealed the following relationships among stressor pile‐up, family satisfaction, relationship satisfaction, and stress:

**TABLE 3:** Regression results for hypothesized model of moderated mediation.

	Consequent									
	M1: Family satisfaction			M2: Relationship satisfaction				Y: Stress		
Antecedent	*B*	SE	*p*	*B*	SE	*p*		*B*	SE	*p*
Stressor pile‐up	0.3875	0.3525	0.2725	−0.1502	0.2888	0.6034		0.3521	0.3195	0.0007
Family satisfaction	–	–	–	–	–	–		0.1881	0.0567	0.0010
Relationship satisfaction	–	–	–	–	–	–		−0.0777	0.0654	0.2359
Family Resilience beliefs (FRES)	–	–	–	–	–	–		−0.2902	0.0871	0.0010
Family satisfaction × FRES	–	–	–	–	–	–		0.2667	0.0986	0.0073
Relationship satisfaction × FRES	–	–	–	–	–	–		−0.1529	0.1777	0.3903
	R2 = 0.0269			R2 = 0.0200				R2 = 0.1836		
	*F* (5, 37,320) = 1.6067			*F* (5, 37,320) = 1.1893				*F* (10, 37,328) = 6.4329**		
	**Effect**	**SE**		**LL 95% CI**	**UL 95% CI**			**Index of moderated mediation**		
Family satisfaction								0.1033, CI [−0.0801, 0.4004]		
Low FRES	0.0359	0.0538		−0.0339	0.1808					
Medium FRES	0.0729	0.0810		−0.0489	0.2728					
High FRES	0.1099	0.1178		−0.0743	0.3906					
Relationship satisfaction								0.0230, CI [−0.1700, 0.4535]		
Low FRES	0.0034	0.0514		−0.1453	0.0740					
Medium FRES	0.0117	0.0409		−0.0718	0.0985					
High FRES	0.0199	0.0806		−0.1085	0.2260					

*Note*: Unstandardized regression coefficients reported. Age, gender, educational attainment and employment status included as covariates. Bootstrap sample = 5000.

Abbreviations: CI, confidence interval; LL, lower limit; UL, upper limit. ** *p* < 0.001

The association between stressor pile‐up and family satisfaction (*B* = 0.3875, *SE* = 0.3525, *p* = 0.2725) was not statistically significant. Similarly, the relationship between stressor pile‐up and relationship satisfaction (*B* = −0.1502, *SE* = 0.2888, *p* = 0.6034) was also non‐significant. However, stressor pile‐up (*B* = 0.3521, *SE* = 0.3195, *p* = 0.0007) was found to be significantly and positively associated with stress, indicating that higher levels of accumulated stressors lead to increased stress.

Family satisfaction did not serve as a predictor variable for stress directly within this model, as indicated by the absence of values in the table. However, when combined with family resilience beliefs (FRES), family satisfaction (*B* = 0.2667, *SE* = 0.0986, *p* = 0.0073) significantly interacted with FRES, indicating that higher family resilience beliefs can moderate the relationship between family satisfaction and stress, potentially mitigating stress levels.

Relationship satisfaction also did not directly predict stress within the model, and the interaction between relationship satisfaction and FRES (*B* = −0.1529, *SE* = 0.1777, *p* = 0.3903) was not statistically significant. This suggests that while relationship satisfaction alone does not have a significant direct effect on stress, it also does not significantly interact with resilience beliefs to affect stress levels.

Interestingly, family satisfaction (*B* = 0.1881, *SE* = 0.0567, *p* = 0.0010) and FRES (*B* = −0.2902, *SE* = 0.0871, *p* = 0.0010) were both significantly associated with stress, implying that higher family satisfaction can reduce stress levels, and stronger family resilience beliefs are associated with lower stress. Conversely, relationship satisfaction (*B* = −0.0777, *SE* = 0.0654, *p* = 0.2359) was not significantly related to stress.

### Moderated mediation analyses

**FIGURE 2:**
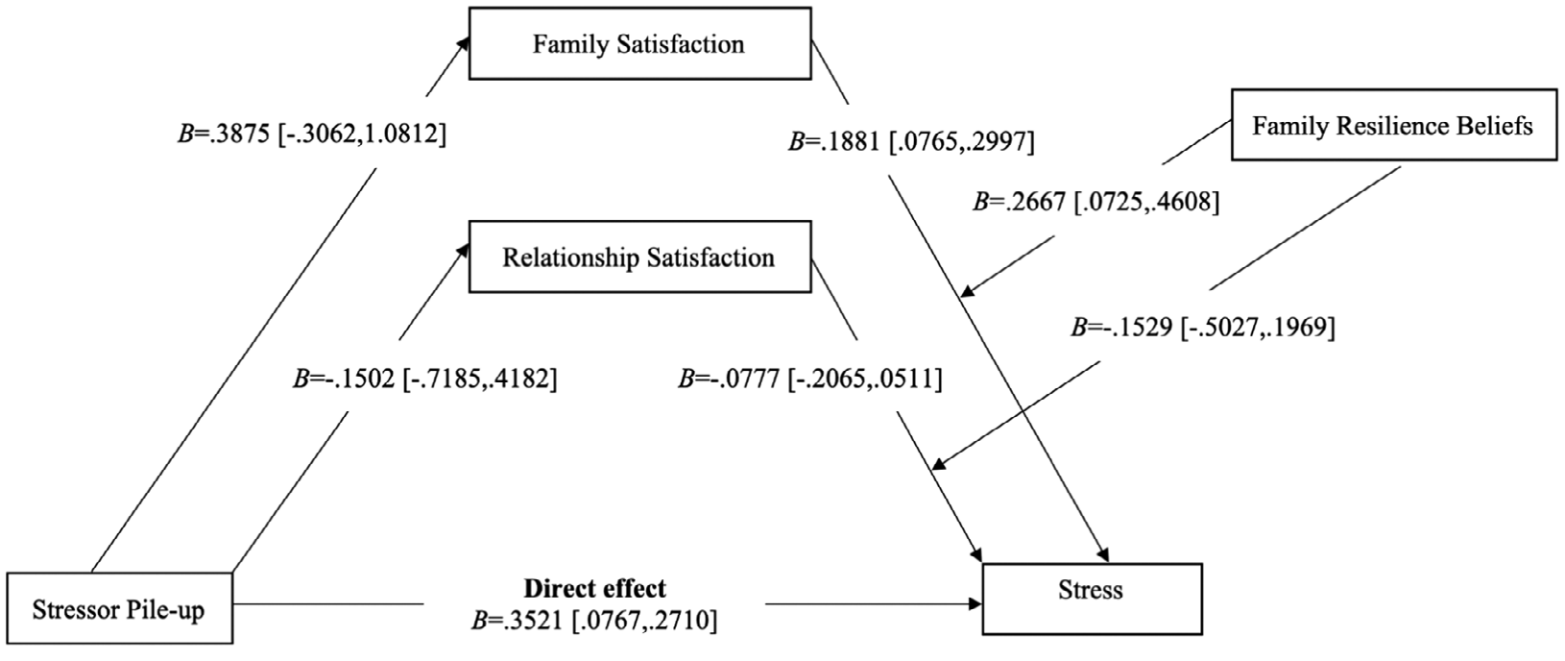
Statistical model.

The hypothesized moderated mediation model was tested using the PROCESS macro model number 14, which tests a model whereby family resilience beliefs moderate the relations of family satisfaction and relationship satisfaction to parent stress (Figure 1; Hayes, 2013). Results of this analysis are presented in Table 3 and depicted visually in Figure 2. The family resilience beliefs moderated the direct associations between family satisfaction and stress (*B* = 0.2677, *SE* = 0.0986, *p* = 0.0073). Moreover, there is no evidence that family resilience beliefs moderated the direct associations between relationship satisfaction and stress (*B* = −0.1529, *SE* = 0.1777, *p* = 0.3903).

**FIGURE 3:**
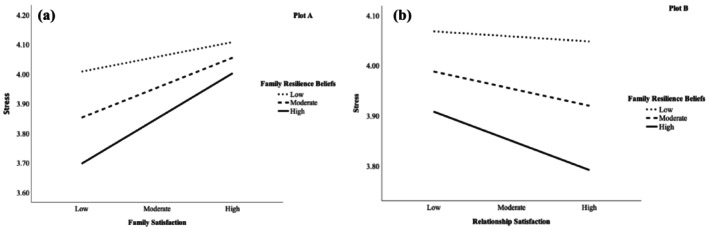
Conditional direct effects of family satisfaction and relationship satisfaction on stress on levels of family resilience beliefs.

Figure 3 presents a visual depiction of the interactions between family satisfaction and family resilience beliefs (plot A) and relationship satisfaction and family resilience beliefs (plot B). Plot A was constructed by estimating the simple effect of family satisfaction on stress scores for low, moderate, and high values of family resilience beliefs. Similarly, plot B was constructed by estimating the simple effect of relationship satisfaction on stress scores for the three levels of family resilience beliefs. As shown in Figure 3 plot A, family satisfaction was positively associated with stress for individuals with all levels of family resilience beliefs, such that as family satisfaction increased, perceived stress increased. As depicted by the steepness of the slopes, the positive relation between family satisfaction and stress among parents was largest in magnitude among with parents with high family resilience beliefs. Figure 3 plot B illustrates that family resilience beliefs moderated the association between relationship satisfaction and stress. Here, results revealed that relationship satisfaction was negatively associated with stress at all levels of family resilience beliefs, with the strongest association for parents with high family resilience beliefs. For parents with high resilience beliefs, higher relationship satisfaction were associated with low stress.

A formal test of moderated mediation based on the index term revealed that the interaction between stressor pile‐up and stress by family satisfaction and relationship satisfaction were positive but non‐significant (Index = 0.1033, 95% CI = −0.0801, 0.4004; Index = 0.0230, 95% CI = −0.1700, 0.4535, respectively), which indicates that family resilience beliefs was a not a moderator of the indirect effect of stressor pile‐up on stress through both family satisfaction and relationship satisfaction. Further hypothesis tests were conducted to determine whether the conditional indirect effect was statistically significant at values corresponding to low (18), moderate (27), and high (36) values of family resilience beliefs. PROCESS automatically generates these conditional indirect effects at moderator values corresponding to the 16th, 50th, and 84th percentile points in the sample data.

There was no evidence that family satisfaction mediated the association between stressor pile‐up and stress for parents with low (effect = 0.0359, CI = −0.0339, 0.1808), moderate (effect =0.0729, CI = −0.0489, 0.2728) or high (effect = 0.1099, CI = −0.0743, 0.3906) family resilience beliefs. Furthermore, relationship satisfaction was not mediating the association between stressor pile‐up and stress for parents with low level (effect = 0.0034, CI = −0.1453, 0.0740); moderate (effect = 0.0117, CI = −0.0718, 0.0985); or high level (effect = 0.0199, CI = −0.1085, 0.2260) of family resilience beliefs. These findings did not confirm our hypothesis, predicting a second stage moderating mediation model where family resiliency beliefs moderated the second stage indirect paths of family satisfaction and relationship satisfaction, creating conditional indirect effects.

## DISCUSSION

More than 4 years since the first documented case of COVID‐19, it is clear that the effects of this public health crisis are far‐reaching and persistent, affecting not only individuals' physical health but also their mental health and overall family well‐being (Brooks et al., 2020; Mazza et al., 2020). The current investigation used data from the large‐scale COVID‐19 Family Life study to investigate personal and relationship‐based influences on European parents' stress symptoms during the early phases of the pandemic. Guided by the ABC‐X theoretical framework, our substantive research question focused on whether the relationship between stressor pile‐up and perceived stress is mediated by two central aspects family functioning (i.e., family satisfaction and relationship satisfaction), and if the proposed mediated relationships are impacted by family resiliency beliefs. The present findings contribute to the research literature by characterizing family mechanisms linking COVID‐19 stressors with parent stress. Moreover, our application of a moderated mediation analytic approach extends prior work by explicitly testing the conditional indirect effects theorized in the ABC‐X conceptual framework among stressor pile‐up, adaptive resources, perception and coherence factors, and adaptive outcomes.

As expected based on prior research, we found a direct association between stressor pile‐up and parents' perceptions of stress, such that parents who reported more pandemic‐related stressors endorsed more symptoms of stress. Around Europe, family life was disrupted in previously unimagined ways as COVID‐19 spread. Families with established routines and reliable social and financial resources suddenly found themselves isolated, forced to assume new responsibilities, fearing for their loved ones health, and dealing with job and income instability. Vulnerable families, such as those with low socio‐economic resources and/or pre‐existing mental and physical health problems were especially challenged by the spread of COVID‐19 and associated mitigation measures. However, given the adequately large sample size of the current study, it is worth noting that the correlation between stressor pile‐up and parental stress is significant yet relatively weak. This finding could be partially explained by the differential susceptibility theory extended by Belsky et al. (2007) based on the diathesis‐stress model. Differential susceptibility theory suggests that some individuals can be more sensitive and susceptible to environmental influences (either positive or negative) due to genetic features (Belsky et al., 2007; Belsky & Pluess, 2009). This theory has been adopted by several scholars to explain individual's adaptive outcomes during the pandemic, yet they mainly focused on youth's stress and developmental outcomes rather than parental stress (e.g., Browne et al., 2021). For the current study, the differential susceptibility theory's proposition might explain the low correlation as for differences in parental stress and adaptation outcomes across these European countries.

Prior research has shown that stressor pile‐up is associated with parental mental health through its effects on family and relationship functioning (Boettcher et al., 2021; Frishman et al., 2017; Manning et al., 2010). Results of the current study, however, did not support a mediating role for either family satisfaction or relationship satisfaction in the link between stressor pile‐up and parents' perceived stress. Contrary to our hypotheses, we did not find direct associations between stressor pile‐up and either family satisfaction or relationship satisfaction. The lack of expected associations may be due to our measurement of stressors. In their seminal work, Randall and Bodenmann (2009) presented a typology of stress within a relational framework. They proposed that impact of stress on relationship satisfaction varies depending on three facets of stressors: the locus of the stressor (external versus internal), the intensity of the stressor (major versus minor stressor), and the duration of the stressor (acute versus chronic stress). The nature of the stressors included in our composite measure of stressor pile‐up are consistent with external stressors – those that originate outside the relationship such as stress from school, work, family members, or social tensions with others outside the relationship (i.e., friends and extended family). Researchers have suggested that to understand the relationships between stress and satisfaction in close relationships, it is critical to study both internal (dyadic) and external stressors individually as well as take into account the interplay between the two with regard to their co‐variation with relationship functioning (Randall & Bodenmann, 2009). For example, the stress‐divorce model posits that external stressors can spillover into the relationship and create internal dyadic stressors (e.g., conflicts and tensions stemming from a decreased sense of mutuality, different goals and needs of partners, worries about the well‐being of a partner). In turn, these internal stressors lead to deterioration of relationship satisfaction (Neff & Karney, 2017; Nguyen et al., 2020). Researchers make a further distinction between major stressor and minor stressors. Major stressors include both normative and non‐normative life events, such as serious illness, accidents, unemployment and death. Minor stressors can also occur across multiple domains of an individuals' life, but encompass the everyday irritating or frustrating demands that present in everyday contact with the environment (e.g., being late to work, forgetting things). Consistent with the ABC‐X theoretical model guiding our work, our measure of stressor pile‐up focused on major life stressors.

Although historically major life stressors have received a great deal of attention in the stress and coping literature, more recent research shows that minor stressors may play an even more important role in understanding relationship functioning. A limitation of our study is that we did not measure family experiences of daily hassles, which may have increased during COVID‐19 because of disrupted family routines, increased parenting duties, and irritations with family members due to close proximity. A recent study bore this out, showing that parents who reported more daily parenting hassles (i.e., minor stressors related to difficulties and irritations related to childcare and education demands in everyday life) had higher depressive, anxiety, and stress symptoms (Li et al., 2022). Together, this research points to complex associations between stress and satisfaction in close relationships. Our specific findings suggest that future studies exploring how stress has affected couple and family relationships during the pandemic should consider both internal and external stressors and focus more on the impact of daily hassles rather than major life stressors.

While our main hypotheses regarding moderated mediation were not supported, we found that the direct association of family satisfaction to parent stress was moderated by family resilience beliefs. The direction of the association, however, was contrary to our expectations informed by prior research. In our sample, family satisfaction was positively associated with stress among individuals with low, moderate, and high levels of family resilience beliefs so that as family satisfaction increased parent stress symptoms also increased. Moreover, the positive relationship between family satisfaction and stress was highest among parents with the highest levels of resiliency beliefs. This puzzling finding is contrary to extant research suggesting a protective effect of resiliency beliefs during times of heightened stress and family disruption (Prime et al., 2020). To interpret this paradoxical finding, we reflect on the novel social and family contexts of the early pandemic. Back then, families were dealing with challenges in balancing work responsibilities and children's educational needs, while shifting from primarily face‐to‐face social interactions with people outside the family to primarily virtual interactions (Kerr et al., 2021; Lee & Chang, 2021). During this uncertain time, parents turned to social media for parenting support, connection, and guidance on how to balance their new responsibilities and keep their children happy and learning in a world where they suddenly were without established support (Drouin et al., 2020). At that time, social media was flooded with ideas about how parents could create daily schedules for children's schooling and activities (e.g., daily exercise time, outdoor time) to make sure their children did not fall behind. Simultaneously, they received implicit and explicit messaging that messages that the extra time at home was ideal for making home improvements and developing new hobbies (Easterbrook‐Smith, 2020). In this context, working parents were also required to meet their employers' expectations of being fully engaged and productive while working from home (Bennett, 2021; Pass & Ridgway, 2022).

Unrealistic pressures to not simply survive, but to thrive, during the early phases of the pandemic may have exacerbated stress for parents. Parents with high resilience beliefs may have felt greater pressure to cope well and demonstrate resilience, compounding the effects of tangible pandemic‐related stressors resulting in increased stress. While additional research is needed to investigate this possibility, a recent study examining parents' lived experiences during the pandemic provides some support for the idea that letting go of expectations for positive adaption may have helped some parents cope better with challenging situational realities (Weaver & Swank, 2020). In addition, other theories could also be helpful to explain the high positive relation between family satisfaction and stress among parents with the highest levels of resiliency beliefs. For instance, the Polyvagal theory proposed by Porges (1995, 2007, 2022) emphasized the role of autonomic nervous system (ANS) in the regulation of stress and human social behaviors. According to this theory, an individual's emotional regulation relies heavily on their social connectedness and feelings of safety, which are supported by the ventral vagal complex (VVC; Porges, 1995, 2007). While introducing and discussing the hierarchy of ANS and its neurophysiological propositions is beyond the current study's scope, the polyvagal perspective (Porges, 2007) has its merits in explaining parental stress adaptation. During the COVID‐19 pandemic, the high family satisfaction might made people rely more on social bonds in the face of adversity and a lack of feelings of safety. However, this stressful environment could have overwhelmed VVC to maintain calm, especially for parents with high resilience beliefs, leading to an increase in stress of meeting high‐demanding expectations in this challenging context, despite high family satisfaction and the probability that these beliefs could promote coping (Porges, 2022). Given social bonds and connectedness are much needed during adversity based on this theory, the current study did not take community‐level factors into account in the analysis even though community resources could be important for European parents' adaptations. Thus, factors such as neurobiological mechanisms, self‐compassion, and psychological flexibility (Coyne et al., 2020) may be more important to consider or foster than resiliency beliefs in crisis contexts.

Relationship satisfaction and family resilience beliefs did not interact in the prediction of parent stress. There was also no significant main effect for relationship satisfaction on parent stress in multivariate models controlling for socio‐demographic covariates (gender, age, educational attainment, and employment status). These results are largely inconsistent with literature documenting an explanatory role for relationship satisfaction in the association between stress and mental health in general (Braithwaite & Holt‐Lunstad, 2017), as well as recent studies finding direct links between relationship satisfaction and partners' mental health during the pandemic (Turliuc & Candel, 2021; Waddell et al., 2021). The lack of association in adjusted models indicates that relationship satisfaction, as measured in the current study, is not independently associated with individuals' perceptions of stress during the pandemic. Although many stressors associated with psychological distress are confounded with couples' relationship satisfaction, studies exploring nuanced aspects of intimate relationship quality have identified specific relational characteristics that demonstrate more robust associations with partners' psychological experiences during times of upheaval. For example, in a recent study researchers examined three sources of relational uncertainty (self‐uncertainty, partner uncertainty, and relationship uncertainty) along with two aspects of interdependence (interference and facilitation) with respect to intimate partners' symptoms psychological distress over time during COVID‐19 (Estlein et al., 2022). Findings pointed to distinctive associations between different sources of relational uncertainty and indices of psychological distress including anxiety, depression, and somatization. Specifically, although relationship uncertainty strongly predicted all three facets of psychological distress, self‐uncertainty was associated with somatization and depression, and partner uncertainty was associated with depression and anxiety. Moreover, only relationship uncertainty predicted psychological distress across all three time points (i.e., the start of lockdown, middle of lockdown, and after lockdown).

### Limitations and future directions

There are several methodological limitations of the present study. First, this is a cross‐sectional investigation and thus prevents interpretations of causality regarding the relationships among study variables. Ongoing monitoring utilizing prospective longitudinal study designs is critical to fully understand the relations between pandemic stressors, family relationships, and mental health outcomes throughout the pandemic and post‐pandemic. Research investigating parental and familial adaptations through across‐subsystem lens could yield meaningful insights, e.g., how communities provide resources for various families could have mitigated their financial strain and offered emotional support. This would help develop prevention programs and targeted interventions to foster positive adaptation in families. Second, convenience sampling with self‐report surveys was used for data collection. While this allowed for a large amount of data to be collected in a short period of time, it introduced external validity concerns as well as biases, including selection bias (e.g., some participants could be harder to be reached), recall bias, and social desirability bias. Third, while a unique aspect of this study is its focus on a large number of countries and large sample size, this does introduce potential problems related to generalizability. Not just cultural diversity and socioeconomic variations, but also differences in COVID‐19 mitigation approaches and overall pandemic impacts during the data collection period across countries may obscure important differences among respondents in the constructs examined. Fourth, data were collected from individuals rather than couples, with precludes our ability to account for reciprocal influences that partners have on each other with regards to factors that impact relationship satisfaction and psychological distress.

## CONCLUSION

Positive attributes of this study included the use of an analytic approach that considered two mediators and one moderator simultaneously, our large sample, and the use of well‐validated questionnaire measures. In general, our findings suggest that the ABC‐X model, and variables selected based on the model, may not have captured important relationship processes that affect parents' experience of stress early in the pandemic. Alternative models, such as the vulnerability‐stress‐adaptation model may better elucidate associations among these variables. Another important direction for future research is closer examination of the potential negative impact of resiliency beliefs on mental health for some parents. Such work should explore additional risk and protective factors, such as self‐compassion, that may affect parental well‐being.

## Supporting information


Table S1.
