# Supplementary material for: Exploring the factors contributing to parent stress symptoms during the COVID‐19 pandemic in Europe: An ABC‐X model approach
Source: Fam Process. 2024 Sep 27;64(1):e13063. doi: 10.1111/famp.13063 (PMC11786249; doi:10.1111/famp.13063)
Supplement: Supplementary file 1 — Table S1. [file FAMP-64-0-s001.docx]

**Supplementary Material**

*Cronbach’s Alpha Values and CFA Model Fit*

| Language | Stressor Pile-Up | Family Satisfaction | Relationship Satisfaction | Family Resilience Beliefs | Parent Stress Symptoms |
| --- | --- | --- | --- | --- | --- |
| English | α = 0.78, CFI = 0.95, TLI = 0.94, RMSEA = 0.06 | α = 0.92, CFI = 0.97, TLI = 0.96, RMSEA = 0.04 | α = 0.88, CFI = 0.95, TLI = 0.94, RMSEA = 0.05 | α = 0.91, CFI = 0.96, TLI = 0.95, RMSEA = 0.05 | α = 0.78, CFI = 0.94, TLI = 0.93, RMSEA = 0.06 |
| German | α = 0.83, CFI = 0.93, TLI = 0.92, RMSEA = 0.07 | α = 0.90, CFI = 0.96, TLI = 0.95, RMSEA = 0.05 | α = 0.85, CFI = 0.94, TLI = 0.93, RMSEA = 0.06 | α = 0.88, CFI = 0.95, TLI = 0.94, RMSEA = 0.05 | α = 0.93, CFI = 0.97, TLI = 0.96, RMSEA = 0.04 |
| French | α = 0.91, CFI = 0.96, TLI = 0.95, RMSEA = 0.05 | α = 0.87, CFI = 0.95, TLI = 0.94, RMSEA = 0.06 | α = 0.94, CFI = 0.98, TLI = 0.97, RMSEA = 0.03 | α = 0.78, CFI = 0.92, TLI = 0.91, RMSEA = 0.07 | α = 0.89, CFI = 0.95, TLI = 0.94, RMSEA = 0.05 |
| Italian | α = 0.86, CFI = 0.94, TLI = 0.93, RMSEA = 0.06 | α = 0.93, CFI = 0.97, TLI = 0.96, RMSEA = 0.04 | α = 0.87, CFI = 0.95, TLI = 0.94, RMSEA = 0.06 | α = 0.90, CFI = 0.96, TLI = 0.95, RMSEA = 0.05 | α = 0.84, CFI = 0.93, TLI = 0.92, RMSEA = 0.07 |
| Spanish | α = 0.88, CFI = 0.95, TLI = 0.94, RMSEA = 0.05 | α = 0.91, CFI = 0.96, TLI = 0.95, RMSEA = 0.05 | α = 0.86, CFI = 0.94, TLI = 0.93, RMSEA = 0.06 | α = 0.89, CFI = 0.95, TLI = 0.94, RMSEA = 0.05 | α = 0.94, CFI = 0.97, TLI = 0.96, RMSEA = 0.04 |
| Portuguese | α = 0.92, CFI = 0.97, TLI = 0.96, RMSEA = 0.04 | α = 0.85, CFI = 0.94, TLI = 0.93, RMSEA = 0.06 | α = 0.90, CFI = 0.96, TLI = 0.95, RMSEA = 0.05 | α = 0.83, CFI = 0.93, TLI = 0.92, RMSEA = 0.07 | α = 0.87, CFI = 0.95, TLI = 0.94, RMSEA = 0.06 |
| Greek | α = 0.85, CFI = 0.94, TLI = 0.93, RMSEA = 0.06 | α = 0.89, CFI = 0.95, TLI = 0.94, RMSEA = 0.05 | α = 0.93, CFI = 0.97, TLI = 0.96, RMSEA = 0.04 | α = 0.86, CFI = 0.94, TLI = 0.93, RMSEA = 0.06 | α = 0.91, CFI = 0.96, TLI = 0.95, RMSEA = 0.05 |
| Dutch | α = 0.90, CFI = 0.96, TLI = 0.95, RMSEA = 0.05 | α = 0.84, CFI = 0.93, TLI = 0.92, RMSEA = 0.07 | α = 0.88, CFI = 0.95, TLI = 0.94, RMSEA = 0.05 | α = 0.94, CFI = 0.97, TLI = 0.96, RMSEA = 0.04 | α = 0.85, CFI = 0.94, TLI = 0.93, RMSEA = 0.06 |
| Polish | α = 0.87, CFI = 0.95, TLI = 0.94, RMSEA = 0.06 | α = 0.94, CFI = 0.98, TLI = 0.97, RMSEA = 0.03 | α = 0.82, CFI = 0.92, TLI = 0.91, RMSEA = 0.07 | α = 0.91, CFI = 0.96, TLI = 0.95, RMSEA = 0.05 | α = 0.88, CFI = 0.95, TLI = 0.94, RMSEA = 0.05 |
| Hungarian | α = 0.84, CFI = 0.93, TLI = 0.92, RMSEA = 0.07 | α = 0.88, CFI = 0.95, TLI = 0.94, RMSEA = 0.05 | α = 0.91, CFI = 0.96, TLI = 0.95, RMSEA = 0.05 | α = 0.85, CFI = 0.94, TLI = 0.93, RMSEA = 0.06 | α = 0.93, CFI = 0.97, TLI = 0.96, RMSEA = 0.04 |
| Romanian | α = 0.93, CFI = 0.97, TLI = 0.96, RMSEA = 0.04 | α = 0.86, CFI = 0.94, TLI = 0.93, RMSEA = 0.06 | α = 0.89, CFI = 0.95, TLI = 0.94, RMSEA = 0.05 | α = 0.87, CFI = 0.95, TLI = 0.94, RMSEA = 0.06 | α = 0.90, CFI = 0.96, TLI = 0.95, RMSEA = 0.05 |
| Croatian | α = 0.86, CFI = 0.94, TLI = 0.93, RMSEA = 0.06 | α = 0.92, CFI = 0.97, TLI = 0.96, RMSEA = 0.04 | α = 0.84, CFI = 0.93, TLI = 0.92, RMSEA = 0.07 | α = 0.93, CFI = 0.97, TLI = 0.96, RMSEA = 0.04 | α = 0.81, CFI = 0.91, TLI = 0.90, RMSEA = 0.08 |
| Estonian | α = 0.89, CFI = 0.95, TLI = 0.94, RMSEA = 0.05 | α = 0.83, CFI = 0.93, TLI = 0.92, RMSEA = 0.07 | α = 0.92, CFI = 0.97, TLI = 0.96, RMSEA = 0.04 | α = 0.88, CFI = 0.95, TLI = 0.94, RMSEA = 0.05 | α = 0.94, CFI = 0.98, TLI = 0.97, RMSEA = 0.03 |
| Lithuanian | α = 0.82, CFI = 0.92, TLI = 0.91, RMSEA = 0.07 | α = 0.91, CFI = 0.96, TLI = 0.95, RMSEA = 0.05 | α = 0.85, CFI = 0.94, TLI = 0.93, RMSEA = 0.06 | α = 0.94, CFI = 0.98, TLI = 0.97, RMSEA = 0.03 | α = 0.87, CFI = 0.95, TLI = 0.94, RMSEA = 0.06 |
| Bulgarian | α = 0.88, CFI = 0.95, TLI = 0.94, RMSEA = 0.05 | α = 0.78, CFI = 0.92, TLI = 0.91, RMSEA = 0.07 | α = 0.94, CFI = 0.97, TLI = 0.96, RMSEA = 0.04 | α = 0.86, CFI = 0.94, TLI = 0.93, RMSEA = 0.06 | α = 0.90, CFI = 0.96, TLI = 0.95, RMSEA = 0.05 |
| Latvian | α = 0.94, CFI = 0.96, TLI = 0.95, RMSEA = 0.05 | α = 0.87, CFI = 0.95, TLI = 0.94, RMSEA = 0.06 | α = 0.78, CFI = 0.93, TLI = 0.92, RMSEA = 0.07 | α = 0.92, CFI = 0.97, TLI = 0.96, RMSEA = 0.04 | α = 0.85, CFI = 0.94, TLI = 0.93, RMSEA = 0.06 |
| Swedish | α = 0.87, CFI = 0.95, TLI = 0.94, RMSEA = 0.06 | α = 0.90, CFI = 0.96, TLI = 0.95, RMSEA = 0.05 | α = 0.86, CFI = 0.94, TLI = 0.93, RMSEA = 0.06 | α = 0.84, CFI = 0.93, TLI = 0.92, RMSEA = 0.07 | α = 0.93, CFI = 0.97, TLI = 0.96, RMSEA = 0.04 |
| Slovak | α = 0.85, CFI = 0.94, TLI = 0.93, RMSEA = 0.06 | α = 0.94, CFI = 0.97, TLI = 0.96, RMSEA = 0.04 | α = 0.89, CFI = 0.95, TLI = 0.94, RMSEA = 0.05 | α = 0.87, CFI = 0.95, TLI = 0.94, RMSEA = 0.06 | α = 0.82, CFI = 0.92, TLI = 0.91, RMSEA = 0.07 |
| Norwegian | α = 0.90, CFI = 0.96, TLI = 0.95, RMSEA = 0.05 | α = 0.84, CFI = 0.93, TLI = 0.92, RMSEA = 0.07 | α = 0.92, CFI = 0.97, TLI = 0.96, RMSEA = 0.04 | α = 0.88, CFI = 0.95, TLI = 0.94, RMSEA = 0.05 | α = 0.86, CFI = 0.94, TLI = 0.93, RMSEA = 0.06 |
| Czech | α = 0.83, CFI = 0.93, TLI = 0.92, RMSEA = 0.07 | α = 0.89, CFI = 0.95, TLI = 0.94, RMSEA = 0.05 | α = 0.87, CFI = 0.95, TLI = 0.94, RMSEA = 0.06 | α = 0.91, CFI = 0.96, TLI = 0.95, RMSEA = 0.05 | α = 0.84, CFI = 0.93, TLI = 0.92, RMSEA = 0.07 |
| Slovene | α = 0.94, CFI = 0.98, TLI = 0.97, RMSEA = 0.03 | α = 0.81, CFI = 0.91, TLI = 0.90, RMSEA = 0.08 | α = 0.88, CFI = 0.95, TLI = 0.94, RMSEA = 0.05 | α = 0.85, CFI = 0.94, TLI = 0.93, RMSEA = 0.06 | α = 0.92, CFI = 0.97, TLI = 0.96, RMSEA = 0.04 |
